# Negative symptoms and speech pauses in youths at clinical high risk for psychosis

**DOI:** 10.1038/s41537-020-00132-1

**Published:** 2021-01-22

**Authors:** Emma R. Stanislawski, Zarina R. Bilgrami, Cansu Sarac, Sahil Garg, Stephen Heisig, Guillermo A. Cecchi, Carla Agurto, Cheryl M. Corcoran

**Affiliations:** 1 Icahn School of Medicine at Mount Sinai, New York, NY USA; 2 IBM T.J. Watson Research Center, Yorktown Heights, NY USA; 3 James J. Peters VA Medical Center, Bronx, NY USA

**Keywords:** Psychosis, Biomarkers

## Abstract

Aberrant pauses are characteristic of schizophrenia and are robustly associated with its negative symptoms. Here, we found that pause behavior was associated with negative symptoms in individuals at clinical high risk (CHR) for psychosis, and with measures of syntactic complexity—phrase length and usage of determiners that introduce clauses—that we previously showed in this same CHR cohort to help comprise a classifier that predicted psychosis. These findings suggest a common impairment in discourse planning and verbal self-monitoring that affects both speech and language, and which is detected in clinical ratings of negative symptoms.

Everyday conversations are governed by a set of rules that are often implicit—taking turns, staying on topic, and using the appropriate volume and tone are required for accurate perception and understanding. One important aspect of communication is pausing. Pauses in speech serve both physiological and cognitive functions. While pausing to breathe is a physiological necessity, cognitive pausing allows speakers not only to plan what they are going to say, but allows their interlocutor a chance to respond^1^. Pauses also hold significant social communicative meaning. The placement and length of a pause can substantially affect the interpretation of an interaction, as longer pauses are often accompanied by negative social attributions^2^.

Abnormalities in speech, including aberrant pausing behavior, are characteristic of schizophrenia and have been examined in the context of negative symptoms. Speech features such as reductions in prosody have been associated with negative symptoms, showing that the most robust speech correlate of negative symptoms in schizophrenia is pause length^3^. While the analysis of linguistic features of spoken language (e.g., semantics and syntax) require transcription and natural language processing algorithms, acoustic qualities of spoken language can easily be recorded and quantified using open-source software which avoids introducing errors through automated transcription. Automated speech analyses have only begun to be applied in clinical high risk (CHR) individuals, with one prior study finding an association between abnormal turn-taking and positive symptom severity^4^. As negative symptoms are prevalent in CHR individuals, we tested the hypothesis that they may be associated with increased time spent pausing, as in schizophrenia. We also tested whether increased time spent pausing was associated with features of syntactic complexity—maximum phrase length and usage of determiner pronouns (that introduce dependent clauses), which themselves were associated with negative symptoms in this same cohort^5^. These syntactic features were identified using automated part-of-speech (POS) tagging^6^ and were input into a machine learning classifier that both predicted psychosis onset previously within this same cohort, and which were associated with negative symptoms in canonical correlation^5^.

CHR individuals had mean total negative symptom ratings of 13.4 (SD 7.8) and mean pause length of 1.0 (SD 0.4) seconds, with mean 48% (SD 16%) of pauses in narrative. Total negative symptom severity was significantly associated with both mean pause length (*r* = 0.64, *p* < 0.005) and percentage of pauses (*r* = 0.60, *p* < 0.005) (Fig. 1), which were themselves intercorrelated (*r* = 0.94, *p* < 0.005). The cohort was stratified by negative symptom severity into subgroups (“low”: 0–1 items >= 3, “medium”: 2–3 items >= 3; “high”: 4–6 items >= 3). These subgroups significantly differed in both mean pause length (F(2,30) = 6.81, *p* = 0.004) and pause percentage (F(2,30) = 6.34, *p* = 0.005), with the “high” subgroup” having significantly longer average pauses (mean 1.32 s (SD 0.44 s) than both the “low” (mean 0.90 s, (SD 0.23 s)) and “medium” (mean 0.84 s, (SD 0.27 s)) negative symptom subgroups. Additionally, the “high” negative symptoms subgroup had a significantly higher pause percentage (mean 59% (SD 15%) than the “low” (mean 40% (SD 15%) and “medium” (mean 42% (SD 10%) negative symptoms subgroups. The linguistic variable of maximum phrase length was negatively associated with both mean pause length (*r* = −0.57, *p* = 0.001) and percentage of pauses (*r* = −0.56, *p* = 0.001), and determiner pronoun usage was negatively associated with average pause length (*r* = −0.37, *p* = 0.03) but not percentage of pauses (*p* > 0.13). Of note, both maximum phrase length (*r* = −0.47, *p* = 0.006) and determiner pronoun use (*r* = −0.39, *p* = 0.03) were significantly negatively correlated with total negative symptoms. In this cohort, there were no associations of age or sex with negative symptoms, mean pause length or percentage of pauses (all *p*’s > 0.30). No group differences were found for pause variables by medication prescription or by total words spoken by the interviewer (all *p*’s > 0.12).

**Fig. 1:**
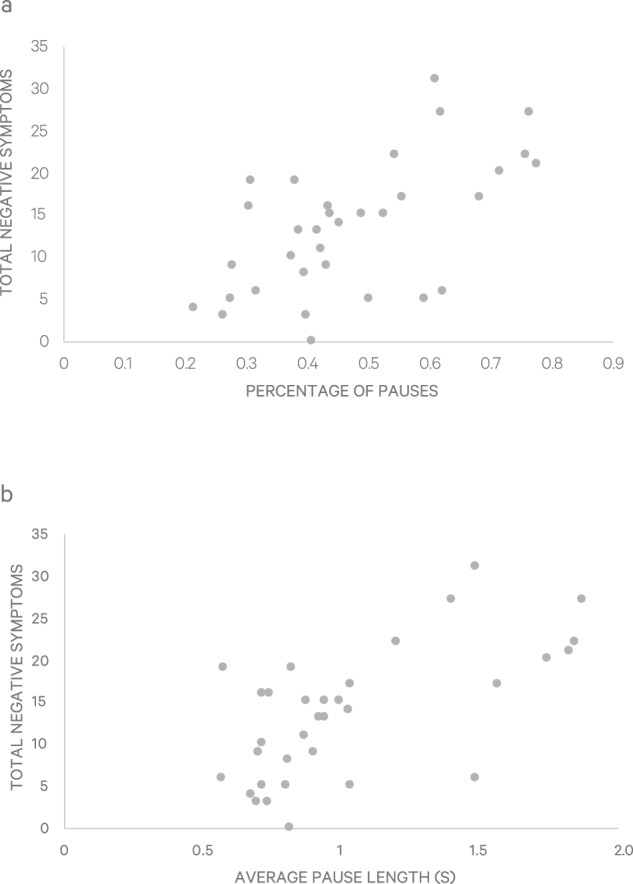
Associations of negative symptom severity and pause behavior. Panel 1**a** shows the association of negative symptom severity with mean pause length in seconds. Panel 1**b** shows the association of negative symptoms severity with percentage of pauses.

Overall, using open-source automated acoustic analysis, longer pause lengths and percentage of time spent in silences (e.g., long pauses) were found to be associated with both negative symptoms and shorter maximum phrase length in individuals at clinical risk for psychosis. These results are consistent with the prior study by Cohen et al.^3^, which identified pause length as the sole speech feature (among seven acoustic variables) that was consistently associated with negative symptom severity in schizophrenia across five studies with disparate methods for soliciting speech, including interviews, as in the current study, but also in social cognition tasks and monologues.

The association between negative symptoms and increased time spent pausing across stages of psychosis might be based in cognitive and linguistic mechanisms. A recent study by Cokal et al.^7^ identified that aberrant pauses in schizophrenia lie at the boundaries of large syntactic units as opposed to within clauses, indicating that word-finding (reflected by within-clause pausing) is not the problem, but instead a difficulty in the organization of thought in speech, which occurs at clausal boundaries. Therefore, it may be that longer pauses reflect impairment in discourse planning and verbal self-monitoring, similar to what has been found for measures of thought disorder^8^. This premise is supported by neuroimaging studies that show attenuation of activity during pauses in regions of the brain involved in the planning and monitoring of speech, specifically left superior temporal gyrus and insula^9^. Further, these pauses may be interpreted clinically as reflecting alogia and diminished emotional expression, as manipulation of pre-recorded patient speech to artificially inflate pause length, holding all other acoustic variables constant, leads to higher ratings of negative symptoms in patients with schizophrenia^10^.

There are a few limitations in this pilot study, namely modest sample size and lack of a comparison group. Future directions will include the study of pauses and other acoustic variables, as well as additional measures of syntactic complexity, in a large prospective psychosis risk cohort study with healthy and patient comparison participants fully characterized in respect to symptoms and cognition, with potential contributions of medications, motivational impairments, and social behavior considered. Finally, mechanistic studies of these putative biomarkers of negative symptoms can identify targets for engagement in preventive intervention strategies.

## Methods

### Participants and clinical characterization

Participants met criteria for the “Clinical High Risk” (CHR) syndrome of attenuated positive symptoms, as assessed with the Structured Interview for Psychosis-Risk Syndromes/Scale of Psychosis-Risk Symptoms^11^. Participants were 33 CHR individuals with mean(SD) age = 21(4) years, who were 1/3 female and ethnically diverse (36% Caucasian). 24% of participants were prescribed medications (9% antipsychotics, 21% antidepressants). Adult participants provided written informed consent; participants under 18 provided written assent, with written consent provided by a parent. This study was approved by the Institutional Review Boards at first the New York State Psychiatric Institute at Columbia University, and then at the Icahn School of Medicine at Mount Sinai. Negative and other symptoms were assessed by PhD raters using the Structured Interview for Psychosis-Risk Syndromes/Scale of Psychosis-Risk Syndromes (SIPS/SOPS)^11^, separate from narrative interviews.

### Speech elicitation

Speech was elicited through open-ended narrative interviews^12^, in which participants were instructed to discuss their lives broadly; interviews were conducted using qualitative methods^13^ meant to maximize the amount of narrative speech by the person interviewed with interviewers interjecting only to encourage the participant to speak further. Interviewers were trained in qualitative interviewing by an expert in phenomenological research methods^8^. Over approximately one hour, participants were encouraged to describe their experience, its impact on them, and their expectations for the future.

### Speech preprocessing and pause analysis

Interviewer speech was manually spliced and removed from transcripts to ensure pauses were only those that could be attributed to the participant. Pause analysis was conducted using PRAAT (www.fon.hum.uva.nl/praat). Based on the criterion set forth by Goldman-Eisler^14^, pauses were defined as any silence longer than 250 ms, as pauses shorter than 250 ms are considered to signify breathing and articulation, while pauses longer than 250 ms are assumed to reflect higher level cognitive processes. Mean pause length was calculated, as well as the percentage of time during the encounter spent in silences greater than 250 ms, defined as the percentage of pauses. These variable types (mean and percentage) were considered to be relatively insensitive to length of audio recordings, which were shorter among individuals with highest negative symptoms (see below); (F(2,30) = 3.80, *p* = 0.03).

### Natural language processing analysis: preprocessing and part-of-speech tagging

Interviews were transcribed by an independent company. Transcripts were preprocessed using the Natural Language Toolkit (NLTK; http://www.nltk.org/). After discarding punctuation, each interview was automatically parsed into phrases. Words were then converted to the roots from which they are inflected, or lemmatized, using the NLTK WordNet lemmatizer. The resultant preprocessed data consisted of a list of lemmatized words, parsed into phrases, maintaining the original order, without punctuation and in lower case. Maximum phrase length and usage of determiner pronouns (which introduce clauses) were determined previously using part-of-speech (POS) tagging, as described in our prior study of linguistic predictors of psychosis onset and linguistic correlates of prodromal symptoms^5^.

### Data analyses

Spearman correlation analyses were done for pause variables with demographics, negative symptoms and two linguistic features of syntactic complexity (maximum phrase length and use of determiner pronouns). Also, following Cohen et al.^3^, participants were stratified by negative symptom severity, here using the SIPS/SOPS, with an enumeration of negative symptoms rated as 3 or higher: low as 0–1 items >= 3, *N* = 9; medium as 2–3 items >= 3; *N* = 12, and high as 4–6 items >= 3, *N* = 12. ANOVA analyses were used to identify differences in pause variables between these subgroups. Alpha was set at 0.05 for hypothesized associations of pause variables with negative symptom severity, and with linguistic variables of syntactic complexity, maximum phrase length, and use of determiner pronouns. No adjustment was made for covariates in this small sample. All tests were two-sided.

### Reporting summary

Further information on experimental design is available in the Nature Research Reporting Summary linked to this article.

## Supplementary information

Reporting Summary

## Data Availability

The data that support the findings of this study are available from the corresponding author upon request.

## Supplementary information

**Supplementary information** The online version of this article (10.1038/s41537-020-00132-1) contains supplementary material, which is available to authorised users.

## Code availability

All analyses were conducted in Praat and Python. The code is available from the corresponding author upon request.
